# Impact of a Maternal Motivational Interviewing on Oral Health in the Mother-Child Dyad

**DOI:** 10.3390/healthcare10061044

**Published:** 2022-06-03

**Authors:** M. Á. Ramírez-Trujillo, M. C. Villanueva-Vilchis, L. A. Gaitán-Cepeda, F. C. Aguilar-Díaz, M. E. Rojas-Russell, J. Fuente-Hernández

**Affiliations:** 1 Department of Public Health, National School of Higher Studies, Leon Unit, National Autonomus University of Mexico, Leon CP 37684, Mexico; mramirez@enes.unam.mx (M.Á.R.-T.); faguilar@enes.unam.mx (F.C.A.-D.); fuente@unam.mx (J.F.-H.); 2 Department of Oral pathology and Oral Medicine, Graduate and Research Division, Dental School, National Autonomous University of Mexico, Mexico City CP 04360, Mexico; lgaitan@unam.mx; 3 School of Higher Studies (F.E.S.) Zaragoza, National Autonomous University of Mexico, Mexico City CP 09230, Mexico; merr@unam.mx

**Keywords:** motivation, health knowledge, attitudes, practice, self-efficacy, health education, mother-child relations, maternal health, infant health, oral health

## Abstract

Motivational Interviewing (MI) has been included in dentistry programs. There exists a need for interventions in the mother-child dyad. The aim of this paper was to compare the effects of a MI-based educational program on oral care knowledge, attitudes, and practices (KAP) in the mother-child dyad to a Traditional Education-based program (TE). A community intervention trial was carried out. The experimental and control groups were made up of women between 18 and 45 years of age in the sixth month of gestation. Both groups were provided with TE. The experimental group additionally received a session based on the principles of the MI. Socio-demographic data, children’s oral health KAP (COHKAP), that of the mother (MOHKAP), and maternal self-efficacy (MSE) in relation to children’s oral health (COHMSE) were recorded. A baseline measurement was made, as well as a six-month follow up. The participants included 135 women with an average age of 24.88 ± 6.00. After intervention, the experimental group’s COHKAP, MOHKAP, and COHMSE all increased (*p* < 0.001). When MI-based interventions are combined with TE, MSE and dental care KAP for the mother-child dyad in pregnant women can be improved.

## 1. Introduction

The health of mothers and children is a high-priority public health issue [1,2]. Since expectant mothers are motivated to adopt behaviors that focus on the care of their newborn offspring, pregnancy is a good time to implement oral health interventions [3,4]. Information on prevention and oral health promotion is generally disseminated via simple strategies such as talks, pamphlets, videos, and other educational materials [5]. These strategies are known as Traditional Education (TE) and have produced very low rates of success [6,7].

Motivational Interviewing (MI) is a collaborative, goal-oriented style of communication with specific attention to the processes of behavior change and has been proposed during the last few decades as a Primary Care method. It is intended to increase personal motivation and commitment to a specific goal by eliciting and exploring the individual’s own reasons for changing behavior in an accepting and compassionate environment [8,9,10]. MI has been applied to the field of health care, including dentistry, and is based on collaboration, evocation, and respect, with a focus on self-efficacy [9,11,12,13,14]. When parents or caregivers use MI, their school-aged children have a lower risk of tooth decay [15] and have more dental checkups and improved oral hygiene [16]. MI prevents tooth decay in children, particularly those from low-income families [17], and increases parents’ oral health knowledge, attitudes, and practices (KAP) for their children’s oral health. However, information on how MI impacts KAPs on oral health of pregnant women and children under five years of age is scarce [5] and inconclusive. Given this, the aim of this quasi-experimental study was to assess the effect of MI during pregnancy on oral healthcare knowledge, attitudes, and practices in the mother-child dyad. Establishing the potential utility of MI in promoting the oral health of the mother and her child will improve population oral health and eventually contribute to the reduction of oral morbidity. 

## 2. Materials and Methods

A quasi-experimental study (six-month blind, parallel, two-group design) was performed at a health care center in León, Guanajuato. The research protocol was approved by the Research Ethics Commission of the institution (CEI.18_013_S1).

### 2.1. Participants

Participants were pregnant women attending prenatal checks at an obstetrics-gynecology care center belonging to the state social security system in the city of León, Guanajuato, Mexico, between July 2019 and January 2020. Women aged 18 to 45 years in their sixth month of pregnancy who had signed an informed consent form were included. Women with a disability or a systemic illness whose pregnancies were declared high-risk, dental professionals, and women with any form of prenatal dental instruction were excluded from the study.

### 2.2. Sample Size

Sample size was calculated using the formula for differences in proportion [18] between mother-child oral care behaviors, contemplating a priori a unilateral difference of 30%, confidence of 95%, and power of 80%, as well as an additional 20% loss on follow-up. This resulted in a total of 38 members in each group.

### 2.3. Allocation

The assay was designed for ten eligible groups. The clusters for the study were groups of 20–23 pregnant women in their sixth month who attended the health service’s “Mothers’ Club”. A number was allocated to each cluster. The first five numbers were assigned to the control group, while the rest were assigned to the experimental group.

### 2.4. Materials 

Sociodemographic data was recorded. The efficacy of the intervention was estimated using the scores of two questionnaires previously validated in this type of population: the Child Oral Health Knowledge, Attitudes, and Practices (COHKAP) composed of 15 items on a scale of 0 to 42 points and a Cronbach’s alpha of 0.82; and the Maternal Oral Health Knowledge, Attitudes, and Practices (MOHKAP) [19]. The child’s oral health maternal self-efficacy was assessed with the Child Oral Health Maternal Self-Efficacy (COHMSE). The MOHKAP and the COHMSE are 10-item questionnaires with scores between 0 and 28 points and a Cronbach’s alpha of 0.79 and 0.93, respectively. A higher score in all three instruments denotes a better expression of the constructs.

### 2.5. Methods

Participants were given a briefing to explain the objectives and the process that would be followed. Their demographic data (age, education, income, marital status, first pregnancy, use of dental services during pregnancy) and baseline data (mothers’ self-efficacy in relation to their babies’ oral health, as well as their knowledge, attitudes, and practices in relation to themselves and their babies) were then recorded. Examiners were blinded and standardized (Kappa = 0.82). The experimental groups were scheduled to attend the next MI session (three weeks later) by the health center social worker. Finally, six months later, a follow-up evaluation was undertaken.

A 15-min theory-practice session for groups of 10–15 persons was offered to deliver the TE, which included infant models and materials appropriate for performing oral hygiene methods for children aged between six months and three years. This program was accompanied by a handout that included information on mouth cleanliness, oral changes, dental appointments, and nutrition for mothers and children. Participants’ phone numbers were taken down at the end of the session, and they were told they would be contacted in two weeks to reinforce what they had learned and respond to any questions. 

The MI group was given the same information and materials as the ET group. The intervention, based on MI principles [9], lasted 20 min in groups of 3–5 women, was given 1 month following the first session. The sessions were led by a credentialed dentist with experience in MI and pediatric dentistry. The agenda was intended to focus on the oral hygiene issues that the women had mentioned, as well as any issues they may have with their children’s oral hygiene. Barriers were recognized and listened to in a reflective manner. Each participant received feedback to ensure that their opinions were properly appreciated. Finally, positive reinforcement and a summary of the issues discussed in the session were presented. Participants were asked for their phone numbers and told they would be contacted in two weeks for a recall call, which was also used to monitor adherence.

### 2.6. Statistical Analysis

Descriptive statistics were calculated for sociodemographic variables. A comparison was made between sociodemographic characteristics and the variables produced by the groups at baseline using Chi-square with Fisher correction. A Wilcoxon test was used to verify the differences before and after the intervention, while the evaluation of the effect of the intervention on the groups was calculated using the Mann-Whitney U Test. Moreover, 2 robust multiple linear regression models were also employed, taking as dependent variables the total COHKAP and MOHKAP scores, in which were included the variables with a significance value of <0.20 in the bivariate analysis.

## 3. Results

### 3.1. Sociodemographic Data

**Figure 1:**
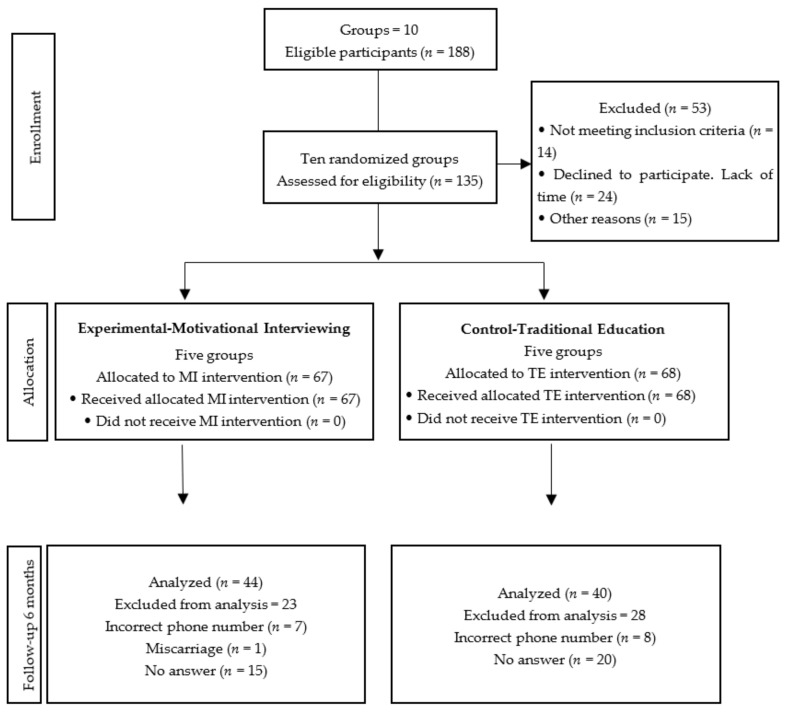
CONSORT flow diagram of assignment of participants from CAISES León, Guanajuato, México, 2019–2020.

The 5 groups assigned to the control group consisted of 68 women while the 5 which also underwent the MI intervention (experimental group) contained 67, leading to a total of 135 expectant mothers at baseline. Follow-up was achieved in 84 (62.2%) women 6 months after the intervention. No women suffered any adverse effects related to participation in the study. The percentage of attrition in the experimental group was 10.2% and 18% for the control. It was found that 68% of these did not answer the phone after 4 successive attempts, 29.41% gave the researcher erroneous phone numbers, and 1.96% had suffered miscarriages (Figure 1).

The average age of the women was 24.88 ± 6.00 with a range of 18 and 41 years. Over half of them reported themselves as solely dedicated to household chores, while half had studied to secondary level. Close to 60% were cohabiting and undergoing their first pregnancies.

There were no significant differences for sociodemographic variables at the baseline between the experimental (*n* = 67) and the control group (*n* = 68) (Table 1).

### 3.2. Baseline Results

At the baseline, the average score on the COHKAP was 31.13 ± 6.18 for the experimental group in the evaluation of maternal oral self-care, and the average score on the MOHKAP was 19.76 ± 4.22 for the experimental group and 18.94 ± 5.14 for the control. There were no significant statistical differences (*p* > 0.05).

Table 2 shows the difference between the two groups on comparing the base measurement with the six-month measurement, along with the differences between the control and experimental groups firstly at the baseline and then in the six-month evaluation.

### 3.3. Follow-up Results

In the follow-up evaluation, for the COHKAP intra-groups, the results show significant statistical differences in the dimensions knowledge (4.15 to 5.11), attitudes (15.25 to 17.38), and practices (11.73 to 13.65). For the MOHKAP, there were significant statistical differences in knowledge (1.97 to 3.25), attitudes (6.28 to 7.22), and practices (11.51 to 13.81). A statistical difference in knowledge variable was found in te control group. There was a significant increase (*p* < 0.001) from the baseline to the follow-up in the total for the two instruments (Table 2).

In the comparison between groups, a difference was noticed in the COHKAP between the control and experimental groups post-intervention (*p* < 0.001), for knowledge the average observed for the experimental group was 5.11 and that for the control was 3.92. For attitudes, these values were 17.38 and 15.17, respectively. In the practices, the value for the experimental group was 13.65 while for the control group was 11.80 (Table 2).

The data on maternal self-care shows that the attitude was the only variable that remained unchanged, with a value of 7.22 for the experimental group compared to 6.35 for the control. (*p* = 0.057) (Table 2). 

### 3.4. Multivariate Results

The robust multiple linear regressions (Table 3) show that belonging to the experimental group increases 3.75 points the child oral health KAP β = 3.75 (IC 95% 1.13–6.37; *p* < 0.001). In the maternal health, the experimental group had an increment of 3.54 points in the MOHKAP scale β = 3.54 (IC 95% 1.78–5.30; *p* < 0.001). Finally, COHKAP showed an increment of 0.52 points for each increment in the COHMSE. No statistical significance was observed between marital status and attendance at psycho-prophylactic courses in the COHKAP analysis, nor any significance relating to marital status, or dental care for MOHKAP.

## 4. Discussion

The aim of this study was to assess the impact of an educational intervention based on MI administered during pregnancy with an evaluation six months afterward, on the oral healthcare knowledge, attitudes, and practices of the mother-child dyad. The results show the usefulness of the MI as a tool for improving the oral health of the mother-child dyad, which signifies a contribution to both the production of data on the MI and an advance in the formulation of health education strategies for disadvantaged social groups.

In this work, the MI administered during pregnancy was associated with an improvement of the KAP of the mother-child dyad. Regarding the knowledge and attitudes of the control group, an improvement was identified in the responses by the mothers to some of the items, regarding the importance of hygiene and visits to the dentist prior to teething, as well as those regarding knowledge of the consequences of dental issues during pregnancy and mothers’ hygiene practices.

This effect in the control group may be due to the fact that these women also received information on these aspects. In addition, the participants were in a favorable clinical environment, and it is also understood that as the due date approaches, there is an improvement in the mothers’ disposition with regard to health care behaviors for their newborns [20].

The dimensions showing the greatest increase in the intervention group were attitudes relating to the child’s oral health (2.13), maternal oral health practices (2.30), and maternal self-efficacy (3.45). Meanwhile, the variables that showed the strongest influence in the comparison between the groups on follow-up after 6 months were attitudes relating the care of the baby (2.21) and maternal care practices (2.29). This leads us to believe that MI has greater potential for modifying attitudes related to the infant’s oral health and those on maternal oral healthcare compared to TE. The results of the regression model indicate that there was an effective impact on the implementation of the intervention based on the MI as well as the positive nature of the maternal self-efficacy, which is considered an essential component in any efforts to achieve improvements in children’s practical oral healthcare habits. It is possible to identify changes in behavior when there is a combination of cognitive and psychosocial aspects, bearing in mind that self-efficacy by itself is no guarantee of good results if the necessary skills and knowledge are lacking. The MI is an approach that increases self-efficacy and reinforces the motivation to change due to its focus on developing healthcare skills [21,22]. The results show that the two components with greatest impact on oral health knowledge, attitudes and practices in the mother-child dyad are the intervention with MI and maternal self-efficacy.

MI is often performed on an individual basis [23]. However, sometimes, time and infrastructure conditions do not allow it. Thus, group implementation has been a strategy to follow and has been implemented before to control alcohol and tobacco consumption [24,25]. Group interventions with MI increase the development of cognitive changes and reduce high-risk behaviors. In addition, this strategy can be reproduced since its affordable and easy to carry out in small spaces. In the context of dentistry, there are few reports of the results of the intervention in a group format that include indicators of change in maternal behavior. The results of this study show a consistent effect on the variables of interest as a positive impact of administering MI, at the group level, for oral health promotion. 

Owing to the COVID-19 pandemic, collecting follow-up data and the inclusion of clinical indices was hindered. In their place, phone surveys were carried out and, although the results reported for both methods are similar, some of the information may have been affected by bias and the social pressure on the participants to answer in a manner they felt was socially desirable. Additionally, this method of data collection results in lower rates of participation compared with face-to-face collection [26]. No online instrument was used in order to avoid any reduction in participation due to lack of internet or multimedia devices among this sector of the population.

Within the limits of the study, it was not possible to carry out individualized randomization. Instead, randomization was applied to those groups with the advantages of greater internal organization, which demonstrated limited individual contamination among their participants. As well as this, some randomized clinical assays require multi-level analysis, which in turn requires large sample sizes and rigorous designs. An analysis of this kind would have been preferable but was not performed as it also would have required a sample size greater than the one planned for [27]. Moreover, it is difficult to recruit a greater number of participants when studying vulnerable populations, such as the one studied here, women in their third trimester of pregnancy. Furthermore, because of the emergence of COVID-19 in Mexico, there was a significant lack of participants at the health service where the study was conducted.

Another limitation of the study is that, although there were no statistically significant differences between the groups in the baseline data, consistently lower values for almost all variables were observed in the control group, which could mean that this group was not dentally motivated in the first instance; therefore, the effect of the intervention could potentially be overestimated.

This study did not consider variables such as psychosocial level, which is known to have a significant influence on healthcare behaviors due to the effects of the stress of motherhood, fatalism, the desire for children, previous losses, attachment issues and postnatal depression, for which reason the expectant mothers were monitored by the CAISES [Essential Health Services Comprehensive Care Center] psychology area. However, these variables definitely ought to be included in future studies dealing with the pre- and postnatal stages.

## 5. Conclusions

This research offered new evidence on the benefits of the MI in terms of enhancing dental health. MI is a promising strategy in the field of dentistry which fosters identification of the consequences, benefits, and ways that behavior can be changed using the resources available in the expectant mother’s sociocultural context. Faithfulness to the spirit of the MI can represent an important means of reducing social inequalities in healthcare.

The new instruments for encouraging self-care should be tailored to the social, cultural, and generational characteristics of the people. The combination of MI and Traditional Educational input generated a discourse in the current study. As a result, the barriers that prevent improved oral health knowledge, attitudes and practices in the mother-infant dyad might be identified.

## Figures and Tables

**Table 1:** Sociodemographic profiles and use of dental services by the expectant mothers commencing and abandoning the study. Leon, Guanajuato, Mexico, 2019–2020.

Participants Commencing the Study					Participants Abandoning the Study			
	MI (*n* = 67)	TE (*n* = 68)	Total (*n* = 135)	*p* ^a^	MI (*n* = 67)	TE (*n* = 68)	Total (*n* = 51)	*p* ^a^
	*n* (%)	*n* (%)	*n* (%)		*n* (%)	*n* (%)	*n* (%)	
Educational level								
Elementary	12 (18)	16 (24)	28 (21)	0.41	1 (4.4)	7 (25)	8 (15.7)	0.11
Secondary	38 (57)	32 (47)	70 (53)		17 (73.9)	11 (39.3)	28 (54.9)	
High school and more	17 (25)	20 (22)	37(27)		5 (21.7)	10 (35.7)	15 (29.4)	
Marital status								
No partner	14 (21)	18 (26)	32 (24)	0.52	4 (17.4)	5 (17.9)	9 (17.6)	0.99
Cohabiting	40 (60)	34 (50)	74 (55)		15 (65.2)	18 (64.2)	33 (64.8)	
Married	13(19)	16 (24)	29 (21)		4 (17.4)	5 (17.9)	9 (17.6)	
First pregnancy								
Yes	31 (46)	41 (60)	72 (53)	0.10	12 (52.2)	15 (53.6)	27(52.9)	0.92
No	36 (54)	27 (40)	63 (47)		11 (47.8)	13 (46.4)	24 (47.1)	
Use of dental services during pregnancy								
Yes	36 (54)	31 (46)	67 (49.6)	0.39	9 (39.1)	15 (53.6)	24 (47.1)	0.40
No	31 (46)	37 (54)	68 (50.4)		14 (60.9)	13 (46.4)	27 (52.9)	
Psychoprophylactic course								
Yes	5 (8)	11 (17)	16 (12)	0.52	0 (0)	4 (14)	4 (8)	0.82
No	62 (92)	57 (83)	119 (88)		23 (100)	24 (86)	47 (92)	

^a.^ Fisher’s exact test.

**Table 2:** Comparison of oral healthcare knowledge, attitudes, and practices between control and experimental groups six months after intervention. CAISES León, Guanajuato, México, 2019–2020.

Variable		Motivational Interview			Traditional Education			Total			Mann Whitney Test
		*n*	Mean	SD ^a^	*n*	Mean	SD	*n*	Mean	SD	*p*
COHKAP	Knowledge BL ^b^	67	4.15	1.85	68	3.87	1.93	135	4.01	1.89	0.34
	Knowledge 6M ^c^	44	5.11	1.16	40	3.92	1.16	84	4.54	1.7	*p* < 0.001
	Wilcoxon	*p* < 0.001			0.83			*p* < 0.001			
	Attitudes BL	67	15.25	3.17	68	14.82	3.99	135	15.04	3.6	0.64
	Attitudes 6M	44	17.38	2.87	40	15.17	3.65	84	16.33	3.43	*p* < 0.001
	Wilcoxon	*p* < 0.001			0.09			*p* < 0.001			
	Practices BL	67	11.73	2.78	68	11.41	3.63	135	11.57	3.23	0.94
	Practices 6M	44	13.65	2.38	40	11.80	3.26	84	12.77	2.97	*p* < 0.001
	Wilcoxon	*p* < 0.001			0.07			*p* < 0.001			
	Total BL	67	31.13	6.18	68	30.10	8.35	135	30.61	7.35	0.61
	Total 6M	44	36.15	5.32	40	30.90	7.79	84	33.65	7.08	*p* < 0.001
	Wilcoxon	*p* < 0.001			0.69			*p* < 0.001			
MOHKAP	Knowledge BL ^b^	67	1.97	1.19	68	2.03	1.19	135	2.00	1.19	0.77
	Knowledge 6M ^c^	44	3.25	0.89	40	2.57	10.5	84	2.92	1.02	*p* < 0.001
	Wilcoxon	*p* < 0.001			0.002			*p* < 0.001			
	Attitudes BL	67	6.28	1.2	68	5.99	2.12	135	6.13	1.72	0.93
	Attitudes 6M	44	7.22	0.85	40	6.35	2.13	84	6.80	1.64	0.057
	Wilcoxon	*p* < 0.001			0.16			*p* < 0.001			
	Practices BL	67	11.51	3.11	68	10.93	2.96	135	11.21	3.04	0.12
	Practices 6M	44	13.81	2.17	40	11.52	2.77	84	12.72	2.71	*p* < 0.001
	Wilcoxon	*p* < 0.001			0.096			*p* < 0.001			
	Total BL	67	19.76	4.22	68	18.94	5.14	135	19.35	4.71	0.28
	Total 6M	44	24.30	2.83	40	20.45	4.82	84	22.46	4.33	*p* < 0.001
	Wilcoxon	*p* < 0.001			*p* < 0.001			*p* < 0.001			

^a^ standard deviation. ^b^ baseline. ^c^ six months follow-up.

**Table 3:** Robust multiple linear regression models. Dependent variables total COHKAP and MOHKAP scores.

Variable		COHKAP				Variable		MOHKAP			
		β ^c^	CI95% ^d^		*p*			β	CI95%		*p*
Intervention	TE ^f^	Ref				Intervention	TE	Ref			
	MI ^e^	3.75	1.13	6.37	0.005		MI	3.54	1.78	5.30	<0.001
Marital Status	No partner	Ref				Marital Status	No partner	Ref			
	With partner	1.16	−1.42	3.74	0.37		With partner	1.06	−0.94	3.06	0.29
Psychoprophylactic course	No	Ref				Use of dental services	No	Ref			
	Yes	−3.10	−7.40	1.19	0.15		Yes	0.92	−0.77	2.61	0.28
MSE ^g^		0.52	0.26	0.78	<0.001						
*p* < 0.001; R-squared = 0.46						*p* = 0.003; R-squared = 0.22					

COHKAP = children’s oral health Knowledge, Attitudes and Practices. MOHKAP = mother’s oral health Knowledge, Attitudes and Practices. ^c^ β = coefficient. ^d^ CI95%: Confidence Interval 95%. ^e^ MI: Motivational Interviewing. ^f^ TE: Traditional Education. ^g^ MSE = maternal self-efficacy in relation to children’s oral health.

## Data Availability

The data sets used and/or analysed during the current study are available from the corresponding author on reasonable request.
